# Neurocognitive and Seizure Outcomes of Selective Amygdalohippocampectomy versus Anterior Temporal Lobectomy for Mesial Temporal Lobe Epilepsy

**DOI:** 10.1155/2014/306382

**Published:** 2014-10-01

**Authors:** Alireza Mansouri, Aria Fallah, Mary Pat McAndrews, Melanie Cohn, Diana Mayor, Danielle Andrade, Peter Carlen, Jose M. del Campo, Peter Tai, Richard A. Wennberg, Taufik A. Valiante

**Affiliations:** ^1^Division of Neurosurgery, University of Toronto, Toronto, ON, Canada M5G 1X8; ^2^Toronto Western Hospital, University Health Network, 399 Bathurst Street, Toronto, ON, Canada M5T 2S8; ^3^Department of Clinical Epidemiology and Biostatistics, McMaster University, Hamilton, Canada L8S 4K1; ^4^Psychology Department, University of Toronto, Toronto, ON, Canada M5T 2S8; ^5^Institute of Medical Sciences, University of Toronto, Toronto, Canada M5T 2S8; ^6^Krembil Neuroscience Center, University Health Network, Toronto, Canada M5T 2S8; ^7^Division of Fundamental Neurobiology, Toronto Western Research Institute, Toronto Western Hospital, Toronto, ON, Canada M5T 2S8; ^8^Department of Neurology, University of Toronto, Toronto, ON, Canada M5T 2S8

## Abstract

*Objective*. To report our institutional seizure and neuropsychological outcomes for a series of patients with mesial temporal lobe epilepsy (mTLE) undergoing anterior temporal lobectomy (ATL) or selective amygdalohippocampectomy (SelAH) between 2004 and 2011.* Methods*. A retrospective study of patients with mTLE was conducted. Seizure outcome was reported using time-to-event analysis. Cognitive outcome was reported using the change principal in component factor scores, one each, for intellectual abilities, visuospatial memory, and verbal memory. The Boston Naming Test was used for naming assessment. Language dominant and nondominant resections were compared separately. Student's *t*-test was used to assess statistical significance.* Results*. Ninety-six patients (75 ATL, 21 SelAH) were included; fifty-four had complete neuropsychological follow-up. Median follow-up was 40.5 months. There was no statistically significant difference in seizure freedom or any of the neuropsychological outcomes, although there was a trend toward greater postoperative decline in naming in the dominant hemisphere group following ATL.* Conclusion*. Seizure and neuropsychological outcomes did not differ for the two surgical approaches which is similar to most prior studies. Given the theoretical possibility of SelAH sparing language function in patients with epilepsy secondary to mesial temporal sclerosis and the limited high-quality evidence creating equipoise, a multicenter randomized clinical trial is warranted.

## 1. Introduction

Anterior temporal lobectomy (ATL) is a well-established and efficacious surgical procedure for the treatment of medically refractory mTLE [1–3]. However, in some patients with mTLE, the ATL procedure has been associated with worsening of cognitive functions, particularly language and memory, when surgery involves the dominant hemisphere [4–7]. Thus, for the appropriate patient population, a more specific resection of the mesial structures through a selective amygdalohippocampectomy (SelAH) has been recommended by some groups [1, 8–10]. The rationale is that SelAH allows for sparing of the nonepileptogenic structures of the neocortex that are potentially involved in language and cognition, potentially resulting in less neuropsychological morbidity [11, 12].

A fair number of studies have been conducted to compare the efficacy of the two surgical approaches with regard to seizure outcome [1, 12–22], neuropsychological outcomes [9, 23–29], or both [10, 30–34] in adults with mTLE. As most of these are retrospective analyses of case series, conclusions are difficult to make given the heterogeneity of pathologies managed, surgical technique, follow-up frequency and duration, neuropsychological test battery, and reporting of seizure outcomes.

Two recent systematic reviews and meta-analyses have been conducted in order to determine the benefit of one procedure over the other with regard to either strictly seizure outcomes [35] or both seizure and neuropsychological outcomes, with the latter focusing on global assessment of intellectual functioning [36]. Importantly, none of the studies included were randomized controlled trials (RCTs) but rather a mix of mainly retrospective and a few prospective studies. Both reviews found that the ATL procedure conferred a higher chance of seizure freedom [35, 36]. Hu and colleagues were not able to identify a statistically significant difference with regard to IQ measurements [36].

As these reviews demonstrate, individual studies may not have sufficient power to detect statistically significant or clinically meaningful differences. However, each can provide valuable data points for meta-analyses. Herein, we seek to contribute the Toronto Western Hospital (TWH) experience, as one of the major sites for adult epilepsy care across Canada. This is a retrospective observational study at TWH, assessing seizure control and neuropsychological outcomes in patients with hippocampal sclerosis as the underlying pathology undergoing either SelAH or ATL, based on a prospectively collected database of a single surgeon (TAV). In all patients examined, cognitive outcome variables that were assessed have been previously validated as sensitive predictors of change in language and cognition [37].

## 2. Methods

### 2.1. Subjects

A retrospective review of a prospectively collected database of patients undergoing SelAH or ATL by a single surgeon (TAV) at the Toronto Western Hospital from January 2004 to December 2011 was conducted. All patients included in the study had medically refractory epilepsy attributable to hippocampal sclerosis, as confirmed on preoperative MRI and postoperative histopathological analysis. All patients included in neuropsychological analyses had complete pre- and postoperative testing. All neuropsychological and seizure evaluations were performed by a consistent team of neuropsychologists and epileptologists, respectively. Additional standard preoperative evaluation included MRI imaging (1.5T scanner, mTLE protocol) and video-EEG through admission to the Epilepsy Monitoring Unit (EMU).

### 2.2. Selection of Surgical Approach

The patients undergoing the SelAH approach were part of a cohort in time, July 2009–July 2010, when our center undertook the SelAH approach in consecutive patients as a trial of a variation to the prior approach of ATL. During this time, patients with MTS but with EEG evidence of neocortical epilepsy were offered ATL while the remainder underwent SelAH. Prior to and after this period, all patients were offered ATL, the standard procedure at our institution for medically refractory mTLE.

### 2.3. Selective Amygdalohippocampectomy

A 2 cm × 0.5 cm corticectomy through the middle temporal gyrus just ventral to the superior temporal sulcus (STS) provides the access window. A subpial approach is utilized following the STS as a plane towards the ventricle. Once the ventricle is entered, the ventricular opening is enlarged. The parahippocampal gyrus is then aspirated in a piecemeal fashion. The hippocampus is removed en bloc. Frameless stereotaxy is subsequently utilized to ensure that the extent of hippocampal resection is to a point behind the tectal plate. The amygdala is resected flush with the roof of the ventricle.

### 2.4. Anterior Temporal Lobectomy

In this approach, the extent of the hippocampal resection is similar to the SelAH with an additional resection of the anterior 4.5 cm or 5.5 cm of the temporal neocortex on the dominant or nondominant side, respectively. The superior temporal gyrus is spared, except the anterior 1 cm. The resection of the amygdala is similar to that of SelAH.

### 2.5. Neuropsychological Testing Battery

Only patients with a full neuropsychological examination prior to and at least 6 months following surgery were included in this component of the analysis. Excluded patients had partial neuropsychological testing which did not allow us to use component measures (e.g., missing verbal component is common in our patients with English as their second language, those who were not seen for postoperative assessment, or individuals who had impaired intellectual functioning assessed as verbal IQ (VIQ) or performance IQ (PIQ) < 70).

Cognitive outcomes were measured using scores from a principal component analysis (PCA), which allows one to reduce data from multiple cognitive measures into single latent components. We have previously demonstrated that these memory PCA scores provide robust estimates of material-specific memory change in mTLE patients; specifically the presurgery verbal memory component predicts postsurgery verbal memory decline, while the presurgery visuospatial memory component predicts visuospatial memory decline [37]. The IQ component score is composed of the Wechsler Abbreviated Scale of Intelligence (WASI), VIQ estimate, and PIQ estimate. The verbal memory component is based on the Rey Auditory Verbal Learning Test (RAVLT) total learning, RAVLT percent retained, and Warrington Recognition Memory Test for words. The visual memory component is composed of Rey Visual Design Learning Test (RVDLT) total learning, Warrington Recognition Memory Test (WRMT) for faces, and Spatial Conditional Associative Learning Test (SCALT) trials to criterion. A detailed method of determining these components has been described previously [37]. In brief, it relies on calculating individual test *z*-scores (based on the distribution of scores in the original patient sample used for the PCA), multiplying each by the latent coefficient associated with the measure, and summing these products to arrive at a PCA score for a particular component.

Visual confrontation naming, shown to be reliably reduced in dominant side ATL, was also tested using the Boston Naming Test (BNT) [38]. The total correct score without phonemic cues was the dependent variable.

### 2.6. Statistical Analysis

All data were analyzed with IBM SPSS Statistics Version 20. Quantitative baseline characteristics were compared between the two groups (ATL or SelAH) using the Student's *t*-test and categorical characteristics were compared using the Chi-squared test.

Student's *t*-test was also used to assess the mean difference for the neuropsychological scores (PCA components and BNT performance) between the two surgical approaches. Analyses were done separately for the dominant and the nondominant groups. Four left-sided temporal lobe epilepsy (TLE) patients had atypical language dominance (one SelAH and three ATL) and were included in the nondominant groups. *P* values less than 0.05 were deemed significant.

Kaplan-Meier curves were generated to determine the relationship between seizure recurrence and the surgical approach. A time-to-event analysis was performed to compare the recurrence rates among the two surgical groups. An “event” was classified as any seizure that occurred after the first postoperative week; seizures occurring in the first postoperative week were excluded. We limited our analysis to a two-year follow-up period to enhance our sample size.

## 3. Results

Overall, 96 patients were included in the study (75 ATL, 21 SelAH); of these, fifty-four (37 ATL, 17 SelAH) completed a full neuropsychological examination prior to and at least 6 months following surgery (median = 11.3 months). Median follow-up was 41 months (range 6–104 months). Patient demographics and a summary of the preoperative evaluations are presented in Table 1. The two groups were well-matched on most variables, except that patients in the SelAH group were slightly younger at the time of surgery and a higher proportion of patients in the ATL group required invasive monitoring prior to surgery (24% versus 5%; Table 1).

**Figure 1:**
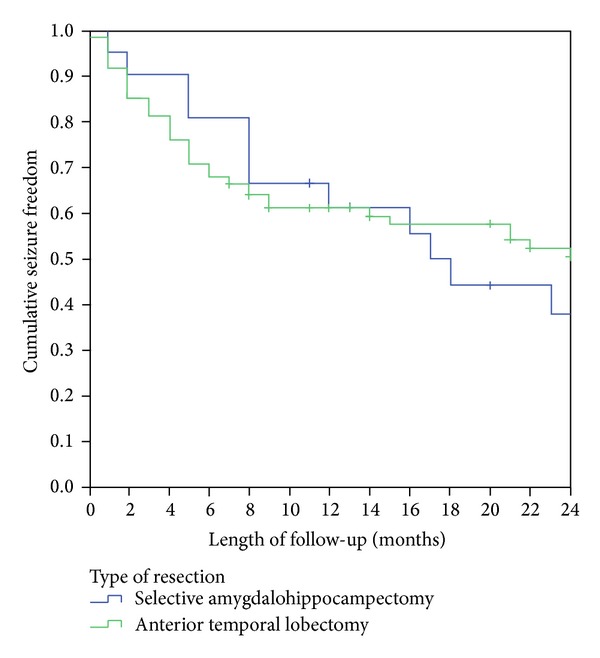
Postoperative seizure freedom in patients with mesial temporal lobe epilepsy as a function of surgical procedure. Analysis time point of two-year follow-up period.

A trend toward earlier seizure recurrence was observed in the ATL group. In the ATL cohort not experiencing early seizures, seizure freedom appeared to be more durable (Figure 1). However, this difference was not statistically significant (HR: 0.85; 95% CI 0.45–1.59; *P* = 0.61).

Invasive EEG (iEEG) monitoring was required in 19 patients; one patient belonging to the SelAH group and the remaining 18 belonging to the ATL group. At our institution, the criteria for iEEG monitoring generally include ictal video EEG findings that are nonconcordant with imaging and neuropsychological examinations, bilateral interictal abnormalities, or multifocal ictal findings. Given that the need for iEEG monitoring is an indication of the complexity of the underlying neuronal network, these patients were more likely to undergo ATL. In patients with bitemporal spikes a standard bitemporal implantation, with hippocampal depth electrodes, in addition to subdural strip electrodes was used. Grid electrodes were used for patients with suspected neocortical involvement. Characteristic differences between the cohort of patients requiring iEEG and those identified as candidates for surgery without iEEG have been outlined in Table 2. While there were no major pre-EMU differences between these two cohorts (outpatient MRI and scalp EEG), a significantly greater proportion of the iEEG cohort was found to have multifocal ictal spikes (*P* < 0.001) and bilateral interictal abnormalities (*P* < 0.001) during video EEG monitoring. While the heterogeneity of these two cohorts suggests a greater complexity with regard to seizure onset localization, there was no statistically significant impact on duration of seizure freedom among them (*P* = 0.08).

Neuropsychological outcomes are shown in Table 3. The PCA change scores can be thought of as “standardized” scores, with a mean of 0 and standard deviation (sd) of 1, based on the distribution of scores in a large population of mTLE patients who have undergone surgery in our centre. They represent postoperative scores minus preoperative scores for each component; a score of  −1.0 indicates a decline in performance that is one sd greater than the normative sample of left and right mTLE surgical cohort and therefore a moderate-to-large decline. Here, none of the PCA change scores differed significantly between the ATL and SelAH groups (all *P* values > 0.3), and all were within one standard deviation of the average change seen in the “normative” sample. Critically for the current hypothesis, there was no systematic advantage seen with respect to memory sparing in the selective procedures; memory was affected equivalently in both groups. Scores for the BNT represent the raw number of items named at postsurgery minus presurgery; again negative scores reflect decline. There was no statistically significant difference amongst groups, although there was a trend for dominant ATL to show a greater decline than other groups (dominant ATL versus nondominant ATL: *t* = 1.58, one-tailed *P* = 0.06; versus nondominant SelAH: *t* = 1.37, one-tailed *P* = 0.09; versus dominant SelAH: *t* = 0.96; one-tailed *P* = 0.20).

## 4. Discussion

The goal of surgery for epilepsy patients is to attain seizure freedom while preventing or minimizing surgical morbidity such as impairments of cognition and memory. As recent meta-analyses suggest, ATL is likely associated with a reduced rate of seizure recurrence relative to SelAH [35, 36]. Given the greater technical challenges of the SelAH procedure [30, 39], it is important to ascertain whether it confers neurocognitive benefits. Both mesial and neocortical temporal lobe structures have roles in acquiring, consolidating, and retrieving material-specific information and the temporal neocortex in the dominant hemisphere is critically involved in naming and other semantic abilities. Given the lack of randomized controlled studies, it is important to consider both seizure and cognitive outcomes in a well-characterized surgical cohort. Furthermore, with respect to the evaluation of cognitive outcomes, validated standardized neuropsychological scores should be included to reduce variability of results.

No statistically significant differences in seizure outcomes were observed in our study. Among studies performed to date, only a few have included patients with hippocampal sclerosis as the sole underlying pathology [15, 21, 23, 25]. Three studies have reported the outcomes of a single surgeon [15, 24, 31], as our study has. Most published series have not identified a significant difference in seizure outcome between the two approaches [23, 26, 30, 31, 34]. Three studies have shown more favorable seizure outcomes in patients undergoing ATL [16, 17, 32]; no studies to date have shown favorable seizure outcomes in patients undergoing SelAH. The meta-analyses of Josephson et al. [35] and Hu et al. [36] demonstrated that ATL conferred an increased likelihood of achieving control from disabling seizures, defined as Engel class I, with an NNT of 10–13 for 1 additional patient to achieve control from seizures (RR 1.32, 95% CI 1.12–1.57) [35]. This benefit was maintained when subgroup analysis was performed on patients who had hippocampal sclerosis as the lone pathology (RR 1.26, 95% confidence interval [1.05–1.51]) [35].

Regarding the question of neuropsychological morbidity, one important aspect of our study was the use of measures that assessed not only IQ, as in several previous studies, but also verbal and visuospatial memory and naming which represent domains that are more specifically related to temporal-lobe function. While we did not observe a statistically significant difference in neuropsychological outcomes between the two procedures, we did observe a trend toward better preservation of naming in patients undergoing SelAH versus ATL on the dominant side. While there are fewer studies that can be amalgamated to evaluate cognitive outcomes and the measures are often heterogeneous, one large-scale series from the Montreal Neurological Institute (123 ATL and 133 SelAH cases) reported that SelAH patients showed better scores compared to patients with ATL in both full-scale and PIQ. Although material-specific memory deficits were apparent following surgery, there were no striking differences between the two approaches [31]. It is possible that there is no clinically meaningful difference between the two approaches with respect to memory measures that are more closely aligned with medial temporal functioning, as was shown by the Montreal Neurological Institute and our results, whereas other cortically mediated functions, such as those reflecting semantic processing, are more likely to show differences.

It is well established that ATL can result in postoperative memory deficits, particularly for verbal memory following dominant hemisphere resection [40]. The magnitude of postoperative decline is strongly influenced by the presence of mesial temporal lobe pathology and the functional integrity of the resected mesial temporal lobe as demonstrated on preoperative memory testing [41–43]. In addition, naming deficits have been documented with the standard ATL on the dominant side [38]. While this is thought to be secondary to resection of relevant functional areas at the temporal base and tip, reduced language comprehension and fluency have been observed in patients undergoing SelAH on the dominant side as well; two narrative reviews [7, 44] reported a greater risk of decline in naming and verbal memory components after resection of the mesial structures on the dominant hemisphere, regardless of the surgical approach. This may reflect deafferentiation in cortical areas, disruption of basal temporal language area pathways, or neocortical lesions secondary to surgical intervention [45]. While there are no meta-analyses directly comparing SelAH and ATL with respect to memory and language, a large number of studies have failed to show a difference in neuropsychological outcomes [23, 24, 26, 28, 46]. However, several studies have reported favorable neuropsychological outcomes in patients undergoing SelAH [10, 12, 18, 19, 25]. Hadar et al. [33] reported an advantage of SelAH in verbal recall based on the RAVLT test, but no difference when overall Wechsler Memory Scale test was used. Goldstein and Polkey found a beneficial effect on immediate verbal recall for paragraphs and verbal paired associate learning for SelAH [4] but could not demonstrate the same results when using Rivermead Behavioral Memory Test to evaluate memory in a more global context [27]. These results open the discussion as to whether the differences on neuropsychological outcomes depend exclusively on the baseline pathology and the surgical approach or whether the specific neuropsychological tests used, and their relative reliance on operations that depend on medial versus neocortical temporal regions, are influential as well.

Whereas the SelAH procedure is restricted to the temporomesial structures [25], ATL involves resections in the dominant (3.5 to 4.0 cm) or nondominant (up to 5 cm) temporal neocortex in combination with an amygdalohippocampectomy [25, 32]. Even in patients with strictly hippocampal sclerosis apparent on preoperative imaging, abnormalities (metabolic, histological, and electrical) have been detected in the temporal neocortex as well [47–49]. Thus, a theoretical advantage of ATL may be attributed to the possible incorporation of epileptogenic foci in addition to the mesial region [35] or by the disconnection of an epileptogenic circuit, preventing seizure propagation and neocortical epileptogenesis. In interpreting the findings in the extant literature, it also must be borne in mind that older studies may not have been as stringent in differentiating mTLE and neocortical TLE, which would erroneously result in a seizure outcome favoring ATL. This is also an important potential confound in considering the lack of compelling differences in cognitive outcome. The expectation is that surgical resection of functionally intact regions should come at a cost. It may be that greater precision in both patient and task selection is required to appropriately estimate the cost associated with resection of the anterior temporal cortex. Even if SelAH can be found to confer an advantage with respect to some aspect of cognitive functioning, there is an argument to be made that patients receiving ATL may fare better over a longer period given the psychosocial benefits derived from improved seizure control [35, 50–52].

Similar to many previous studies, our analysis was limited by its retrospective, nonrandomized nature. In addition, the SelAH procedure was performed within a one-year period; the presence of a learning curve effect could potentially affect outcomes. Furthermore, the database included a greater number of ATL patients. Nonetheless, our findings have contributed a relatively large sample of patients subjected to robust neuropsychological analyses and assessed with stringent seizure recurrence criteria. In addition, our study has the advantage of incorporating data from the experience of a single surgeon, with a homogenous cohort limited only to patients with mTLE with evidence of hippocampal sclerosis. Furthermore, our neuropsychological assessment has implemented data reduction techniques to derive composite scores from a uniform battery of tests pre- and postoperatively. As was demonstrated by St-Laurent et al. [37], this technique has an important value for simplifying measurements while remaining sensitive enough to detect group differences and predict postoperative changes.

Recently, the interest in comparing the two surgical approaches has been rekindled [35] and multicenter randomized control trial (RCT) has been recommended. This is particularly important given that the evidence in favor of ATL for seizure control is growing while the evidence for SelAH for better neuropsychological outcomes remains equivocal. The optimal RCT would be one whereby both seizure and neuropsychological outcomes are assessed. Logistically, however, it may be difficult to obtain a large enough sample that is powered to assess for both outcomes in both dominant and nondominant resections in a single surgical centre. Given that most studies favoring SelAH for neuropsychological outcomes have found that patients with dominant side resections fare better [4, 19], we would advocate for randomization of patients undergoing dominant lobe resections only. Furthermore, it is necessary to establish a unified surgical approach for each procedure and all patients must undergo a standard battery of pre- and postoperative neuropsychological assessments that include measures known to be sensitive to anterior temporal neocortex dysfunction (e.g., naming and semantic fluency) as well as verbal memory. In addition, a standard definition of seizure recurrence and method of analysis must be established and follow-up periods must be predefined. As practice is variable across and within centres, only such Level 1 evidence is appropriate to guide surgical decision-making in weighing seizure and cognitive outcomes of epilepsy surgery.

## Figures and Tables

**Table 1:** Patient demographics and preoperative evaluation.

	SelAH (*n* = 21)	ATL (*n* = 75)	*P* value
Gender (%female)	57	53	0.76
Age at first seizure (Yr)	16.1	13.9	0.52
Age at surgery (Yr)	36	41.9	0.03
History of febrile seizures (%)	6 (29%)	39 (52%)	0.06
Freq. of seizures at time of surgery (per month)	8	9.7	0.40
On multiple AEDs (%)	19 (90%)	62 (83%)	0.60
Initial EEG lateralizing (%)	13 (60%)	45 (60%)	0.99
Contralateral ictal propagation in EMU (%)	3 (14%)	12 (16%)	0.85
Bilateral interictal abnormalities in EMU (%)	3 (14%)	21 (28%)	0.20
Invasive recording needed (%)	1 (5%)	18 (24%)	0.05

*P* values < 0.05 were considered significant.

AED: Antiepileptic drug; EMU: Epilepsy Monitoring Unit.

**Table 2:** Characteristics of patients requiring invasive EEG monitoring.

	Invasive EEG required	Invasive EEG not required
Frequency	19	77
SelAH procedure	1	20
ATL procedure	18	57
Gender (%female)	9 (47%)	45 (58%)
Age at surgery (Year)	37.2	40.9
Initial EEG lateralizing/localizing (%)	9 (47%)	47 (62%)
MRI concordance	14 (72%)	67 (87%)
Multifocal spikes in EMU∗	18 (95%)	19 (25%)
Bilateral interictal abnormalities in EMU (%)∗	9 (47%)	13 (17%)
Average duration of seizure freedom (months)^∧^	18.1	13.7

*P < 0.05.

^∧^ *P* = 0.08.

EEG: electroencephalography; EMU: Epilepsy Monitoring Unit.

**Table 3:** Neuropsychological outcome comparison between dominant and nondominant SelAH and ATL surgical groups.

	Dominant SelAH (*n* = 8)	Dominant ATL (*n* = 12)	Nondominant SelAH (*n* = 9)	Nondominant ATL (*n* = 25)
Change verbal memory PC	−0.7 (1.3)	−0.5 (0.8)	0.2 (0.5)	0.4 (0.7)
Change visuospatial memory PC	0.2 (0.6)	0.3 (0.8)	0.1 (1.2)	0.3 (1.0)
Change IQ PC	−0.4 (0.9)	0.0 (0.8)	−0.2 (0.7)	0.1 (1.0)
Change BNT	−1.0 (7.0)	−4.0 (8.6)	0.1 (2.7)	−0.6 (4.7)

Note: change in BNT for the dominant ATL group is based on *N* = 11. PC: principal component score; BNT is postop-preop total score. Mean and standard deviation for PC and naming scores; no differences are observed between SelAH and ATL in the dominant groups or in the nondominant groups (*P* > 0.31).

## References

[1] Clusmann H, Schramm J, Kral T (2002). Prognostic factors and outcome after different types of resection for temporal lobe epilepsy. *Journal of Neurosurgery*.

[2] Engel JJ, van Ness PC, Rasmussen TB, Ojemann LM, Engel J (1993). Outcome with respect to eplileptic seizures. *Surgical Treatment of the Epilepsies*.

[3] Wolf RL, Ivnik RJ, Hirschorn KA, Sharbrough FW, Cascino GD, Marsh WR (1993). Neurocognitive efficiency following left temporal lobectomy: standard versus limited resection. *Journal of Neurosurgery*.

[4] Goldstein LH, Polkey CE (1993). Short-term cognitive changes after unilateral temporal lobectomy or unilateral amygdalo-hippocampectomy for the relief of temporal lobe epilepsy. *Journal of Neurology, Neurosurgery & Psychiatry*.

[5] Baxendale S, Thompson P, Harkness W, Duncan J (2006). Predicting memory decline following epilepsy surgery: a multivariate approach. *Epilepsia*.

[6] Jones-Gotman M, Zatorre RJ, Olivier A (1997). Learning and retention of words and designs following excision from medial or lateral temporal lobe structures. *Neuropsychologia*.

[7] Sherman EMS, Wiebe S, Fay-Mcclymont TB (2011). Neuropsychological outcomes after epilepsy surgery: systematic review and pooled estimates. *Epilepsia*.

[8] Yaşargil MG, Wieser HG, Valavanis A, von Ammon K, Roth P (1993). Surgery and results of selective amygdala-hippocampectomy in one hundred patients with nonlesional limbic epilepsy. *Neurosurgery Clinics of North America*.

[9] Helmstaedter C, Elger CE (1996). Cognitive consequences of two-thirds anterior temporal lobectomy on verbal memory in 144 patients: a three-month follow-up study. *Epilepsia*.

[10] Pauli E, Pickel S, Schulemann H, Buchfelder M, Stefan H (1999). Neuropsychologic findings depending on the type of the resection in temporal lobe epilepsy. *Advances in Neurology*.

[11] Niemeyer P, Baldwin M, Bailey P (1958). The transventricular amygdala-hippocampectomy in temporal lobe epilepsy. *Temporal Lobe Epilepsy*.

[12] Yaşargil MG, Teddy PJ, Roth P (1985). Selective amygdalo-hippocampectomy. Operative anatomy and surgical technique. *Advances and Technical Standards in Neurosurgery*.

[13] Arruda F, Cendes F, Andermann F (1996). Mesial atrophy and outcome after amygdalohippocampectomy or temporal lobe removal. *Annals of Neurology*.

[14] Paglioli E, Palmini A, Da Costa JC (2004). Survival analysis of the surgical outcome of temporal lobe epilepsy due to hippocampal sclerosis. *Epilepsia*.

[15] Tanriverdi T, Olivier A, Poulin N, Andermann F, Dubeau F (2008). Long-term seizure outcome after mesial temporal lobe epilepsy surgery: corticalamygdalohippocampectomy versus selective amygdalohippocampectomy. *Journal of Neurosurgery*.

[16] Bate H, Eldridge P, Varma T, Wieshmann UC (2007). The seizure outcome after amygdalohippocampectomy and temporal lobectomy. *European Journal of Neurology*.

[17] Mackenzie RA, Matheson J, Ellis M, Klamus J (1997). Selective versus non-selective temporal lobe surgery for epilepsy. *Journal of Clinical Neuroscience*.

[18] Paglioli E, Palmini A, Portuguez M (2006). Seizure and memory outcome following temporal lobe surgery: selective compared with nonselective approaches for hippocampal sclerosis. *Journal of Neurosurgery*.

[19] Renowden SA, Matkovic Z, Adams CBT (1995). Selective amygdalohippocampectomy for hippocampal sclerosis: postoperative MR appearance. *The American Journal of Neuroradiology*.

[20] Sagher O (2013). Epilepsy surgery. *Journal of Neurosurgery*.

[21] Wendling A-S, Hirsch E, Wisniewski I (2013). Selective amygdalohippocampectomy versus standard temporal lobectomy in patients with mesial temporal lobe epilepsy and unilateral hippocampal sclerosis. *Epilepsy Research*.

[22] Schijns OE, Bien CG, Majores M (2011). Presence of temporal gray-white matter abnormalities does not influence epilepsy surgery outcome in temporal lobe epilepsy with hippocampal sclerosis. *Neurosurgery*.

[23] Tanriverdi T, Olivier A (2007). Cognitive changes after unilateral cortico-amygdalo-hippocampectomy or unilateral selective-amyg-dalohippocampectomy for mesial temporal lobe epilepsy. *Turkish Neurosurgery*.

[24] Shin M-S, Lee S, Seol S-H (2009). Changes in neuropsychological functioning following temporal lobectomy in patients with temporal lobe epilepsy. *Neurological Research*.

[25] Helmstaedter C, Richter S, Röske S, Oltmanns F, Schramm J, Lehmann TN (2008). Differential effects of temporal pole resection with amygdalohippocampectomy versus selective amygdalohippocampectomy on material-specific memory in patients with mesial temporal lobe epilepsy. *Epilepsia*.

[26] Lee T, Mackenzie RA, Walker AJ, Matheson JM, Sachdev P (1997). Effects of left temporal lobectomy and amygdalohippocampectomy on memory. *Journal of Clinical Neuroscience*.

[27] Goldstein LH, Polkey CE (1992). Behavioural memory after temporal lobectomy or amygdalo-hippocampectomy. *The British Journal of Clinical Psychology*.

[28] Lacruz ME, Alarcón G, Akanuma N (2004). Neuropsychological effects associated with temporal lobectomy and amygdalohippocampectomy depending on Wada test failure. *Journal of Neurology, Neurosurgery and Psychiatry*.

[29] Helmstaedter C, Reuber M, Elger CC (2002). Interaction of cognitive aging and memory deficits related to epilepsy surgery. *Annals of Neurology*.

[30] Morino M, Uda T, Naito K (2006). Comparison of neuropsychological outcomes after selective amygdalohippocampectomy versus anterior temporal lobectomy. *Epilepsy and Behavior*.

[31] Tanriverdi T, Dudley RWR, Hasan A (2010). Memory outcome after temporal lobe epilepsy surgery: corticoamygdalohippocampectomy versus selective amygdalohippocampectomy. *Journal of Neurosurgery*.

[32] Clusmann H, Kral T, Gleissner U (2004). Analysis of different types of resection for pediatric patients with temporal lobe epilepsy. *Neurosurgery*.

[33] Hadar E, Bingaman W, Foldvary M, Chelune GJ, Comair YG Prospective analysis of outcome after selective amygdalohippocampectomy and anterior temporal lobectomy for refractory epilepsy.

[34] Grivas A, Schramm J, Kral T (2006). Surgical treatment for refractory temporal lobe epilepsy in the elderly: seizure outcome and neuropsychological sequels compared with a younger cohort. *Epilepsia*.

[35] Josephson CB, Dykeman J, Fiest KM (2013). Systematic review and meta-analysis of standard vs selective temporal lobe epilepsy surgery. *Neurology*.

[36] Hu W-H, Zhang C, Zhang K, Meng F-G, Chen N, Zhang J-G (2013). Selective amygdalohippocampectomy versus anterior temporal lobectomy in the management of mesial temporal lobe epilepsy: a meta-analysis of comparative studies a systematic review. *Journal of Neurosurgery*.

[37] St-Laurent M, McCormick C, Cohn M, Mišić B, Giannoylis I, McAndrews MP (2014). Using multivariate data reduction to predict postsurgery memory decline in patients with mesial temporal lobe epilepsy. *Epilepsy and Behavior*.

[38] Ives-Deliperi VL, Butler JT (2012). Naming outcomes of anterior temporal lobectomy in epilepsy patients: a systematic review of the literature. *Epilepsy and Behavior*.

[39] Adada B (2008). Selective amygdalohippocampectomy via the transsylvian approach. *Neurosurgical Focus*.

[40] Bell BD, Giovagnoli AR (2008). Memory after temporal lobe epilepsy surgery: risk and reward. *Neurology*.

[41] Chelune GJ (1995). Hippocampal adequacy versus functional reserve: predicting memory functions following temporal lobectomy. *Archives of Clinical Neuropsychology*.

[42] Stroup E, Langfitt J, Berg M, McDermott M, Pilcher W, Como P (2003). Predicting verbal memory decline following anterior temporal lobectomy (ATL). *Neurology*.

[43] Elshorst N, Pohlmann-Eden B, Horstmann S, Schulz R, Woermann F, McAndrews MP (2009). Postoperative memory prediction in left temporal lobe epilepsy: the Wada test is of no added value to preoperative neuropsychological assessment and MRI. *Epilepsy and Behavior*.

[44] Schramm J (2008). Temporal lobe epilepsy surgery and the quest for optimal extent of resection: a review. *Epilepsia*.

[45] Bartha L, Trinka E, Ortler M (2004). Linguistic deficits following left selective amygdalohippocampectomy: a prospective study. *Epilepsy and Behavior*.

[46] Helmstaedter C, Hufnagel A, Elger CE (1992). Seizures during cognitive testing in patients with temporal lobe epilepsy: possibility of seizure induction by cognitive activation. *Epilepsia*.

[47] Cheung M-C, Chan AS, Lam JMK, Chan Y-L (2009). Pre- and postoperative fMRI and clinical memory performance in temporal lobe epilepsy. *Journal of Neurology, Neurosurgery and Psychiatry*.

[48] Chabardès S, Kahane P, Minotti L (2005). The temporopolar cortex plays a pivotal role in temporal lobe seizures. *Brain*.

[49] Fountas KN, Tsougos I, Gotsis ED, Giannakodimos S, Smith JR, Kapsalaki EZ (2012). Temporal pole proton preoperative magnetic resonance spectroscopy in patients undergoing surgery for mesial temporal sclerosis. *Neurosurgical Focus*.

[50] Elger CE, Helmstaedter C, Kurthen M (2004). Chronic epilepsy and cognition. *The Lancet Neurology*.

[51] Jacoby A, Baker GA, Steen N, Potts P, Chadwick DW (1996). The clinical course of epilepsy and its psychosocial correlates: findings from a U.K. community study. *Epilepsia*.

[52] Helmstaedter C, Kurthen M, Lux S, Reuber M, Elger CE (2003). Chronic epilepsy and cognition: a longitudinal study in temporal lobe epilepsy. *Annals of Neurology*.

